# Predictors of Learning Engagement in the Context of Online Learning During the COVID-19 Pandemic

**DOI:** 10.3389/fpsyg.2022.867122

**Published:** 2022-04-29

**Authors:** Maria Magdalena Stan, Ioana Roxana Topală, Daniela Veronica Necşoi, Ana-Maria Cazan

**Affiliations:** ^1^Faculty of Education, Social Sciences and Psychology, University of Piteşti, Piteşti, Romania; ^2^Faculty of Psychology and Education Sciences, Transilvania University of Braşov, Braşov, Romania

**Keywords:** online learning, learning engagement, online self-efficacy, learning adaptability, self-regulated learning strategies, sources of stress

## Abstract

The main aim of the present research is to analyze the predictive value of individual characteristics such as online self-efficacy, adaptability to uncertainty, and sources of stress during online learning on learning engagement. We also aimed to highlight if these relationships could be mediated by the online self-regulated learning strategies, during the COVID-19 pandemic. The participants were 529 university students and the design was cross-sectional. The results showed significant associations of the sources of stress in online learning with self-efficacy, leaning engagement and self-regulated learning strategies. Self-regulated strategies—task strategies and goal setting represent mediators of stressors perceived by the students under the conditions of the sudden shift to online activity and online learning engagement. The most relevant self-regulation strategies seemed to be goal setting and task strategies, which confirm the need for a clear structure of learning in the context of online activities. The implications of this study reside in the increased awareness regarding how learning engagement in online learning can be predicted by individual characteristics.

## Introduction

In the context of the COVID-19 pandemic, which forced much if not all academic experiences to move to a virtual environment, exploring the possible antecedents of students’ engagement in online learning became a focal point of attention for teachers and researchers. The sudden change in the learning environment has differently influenced the way students engage in learning. For some of them, change was perceived as a challenge, getting involved in academic tasks by showing willingness to invest effort, through concentration and perseverance, even in difficult conditions (e.g., technical difficulties). As we acknowledge that it does tend to show particular features in an online setting, learning engagement remains a strong factor in predicting academic performance (Finn and Zimmer, 2012), positively associated with students’ wellbeing (Asher Steven and Weeks, 2011), and significantly correlated with individual characteristics such as self-efficacy (Reeve and Lee, 2014) and learning adaptability—stemming from Ployhart and Bliese (2006). I-ADAPT theory which identifies individual adaptability as “an individual’s ability, skill, disposition, willingness, and/or motivation, to change or fit different task, social, and environmental features” (p. 13). Learning adaptability refers to one’s approach toward change with regard to learning challenges—as a form of self-regulation in challenging contexts (Collie and Martin, 2017). Although many studies on online learning before the pandemic tried to understand the factors influencing student’s engagement in online learning, the COVID-19 crisis demanded educators and students to suddenly adjust to an unplanned situation, the remote teaching and learning context, which differ from the classic online learning. Therefore, the main aim of the present research is to analyze the predictive value of individual characteristics such as online self-efficacy, adaptability to uncertainty, and sources of stress during online learning on learning engagement. We also aimed to highlight if these relationships could be mediated by the online self-regulated learning strategies, during the COVID-19 pandemic.

## Literature Review

### Online Learning and Learning Engagement. Antecedents of Learning Engagement

Learning engagement is a key piece in trying to understand students’ academic behavior. The growing research interest surrounding learning engagement is well justified. As Kuh (2009) simply put it “higher engagement levels and higher grades go hand-in-hand” (p. 11), as engaged academic behavior may be a powerful predictor for performance. The construct builds heavily upon theories such as Astin (1999), which discusses student involvement as “the quantity and quality of the physical and psychological energy that students invest in the college experience” (p. 528), and (Schaufeli et al., 2002) which define engagement as a “positive, fulfilling, work-related state of mind that is characterized by vigor, dedication, and absorption” (p. 74). On the same note, Appleton et al. (2006) describe academic engagement as a multifaceted construct, an attitude-like, ABC structural model approach (Eagly and Chaiken, 1998) to the academic experience, entailing: an affective dimension (feelings of identification or belonging, and relationships with teachers and peers—for psychological engagement), a behavioral dimension (time on task, credits earned toward graduation, and homework completion, while attendance, suspensions, voluntary classroom participation, and extracurricular participation) and a cognitive dimension (self-regulation, relevance of schoolwork to future endeavors, value of learning, and personal goals and autonomy—for cognitive engagement).

As core part of the overall academic experience, learning engagement can be considered students’ willingness and behavioral commitment to participate in learning activities related to their academic role. Therefore, commitment to educational goals (Tinto, 2017), willingness to engage based on specific beliefs about oneself and other education related variables, becomes an important trigger, leading to actions (learning actions) that students take, in a continuing form, toward achieving these goals. Analyzing relevant correlations, learning engagement has been significantly linked to academic achievement (Carini et al., 2006; Kuh, 2009; Loscalzo and Giannini, 2019; García-Martínez et al., 2021), low levels of psychological distress (Schaufeli et al., 2002), increased educational persistence (Tinto, 2017) and satisfaction with student experience (Truta et al., 2018; Kandiko Howson and Matos, 2021).

Student engagement refers to behavioral (incorporating participation, effort, persistence, and positive conduct), emotional (interactions with teachers and classmates and a sense of belonging) and cognitive dimensions, including self-regulated learning (Fredricks et al., 2016). Knowing that the presence of strong relationships between students and teachers has emerged as a crucial factor in promoting student engagement, it is obvious that the remote learning context could negatively affect student engagement, school closure being an important barrier to engagement, as previous studies also showed (Devitt et al., 2020). The literature on this topic highlights several factors influencing students’ engagement in online learning. Social support and more specifically teacher presence and support are important factors that positively influence students’ engagement (Ansong et al., 2017; Nortvig et al., 2018). In a study regarding students’ perception on what’s missing in online learning, Stodel et al. (2006) found that many of the participants were first-time online learners which led them to experience anxiety related to online learning, feeling concerned about performing online tasks and being on the right track. Another interesting finding from Stodel et al. (2006) study was that, when in an online setting, learners miss social presence the most, showing that social presence is a crucial factor of engagement.

Well-designed and structured online content and activities enhance student engagement in the learning process (Zydney et al., 2020). In a study regarding students’ engagement in online learning environment, using Archer (2003) and Kahn et al. (2017) found that learners become engaged in the academic online activities through a process of reflexive deliberation, students seeking to establish concrete courses of action and sustained practices in the face of uncertainty and complexity (Kahn et al., 2017). As students face uncertain times and changing learning settings, well-structured courses and activities could enhance student engagement in the learning process.

**FIGURE 1:**
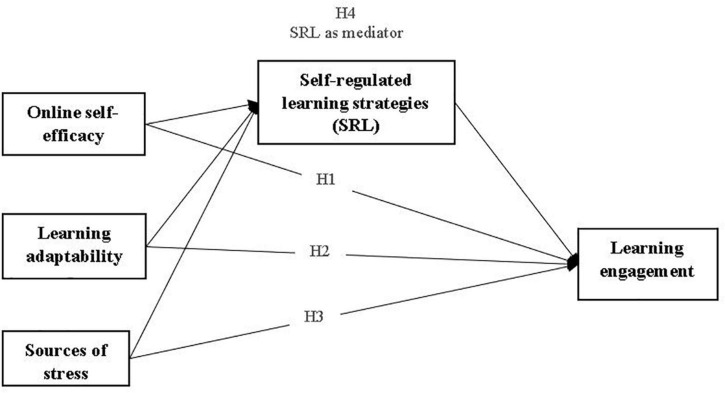
The research model.

Previous experience with technology and technology self-efficacy are also discussed as factors with positive effects on engagement in online learning (Chen, 2017; Lie et al., 2020). Research on online learning and self-efficacy refers to student’s confidence in their ability of using online resources and other related technology. Research shows that previous experience can predict completion rates in online distance education courses (Jan, 2015), computer and online self-efficacy being higher for students who had prior training on computers or who had previously taken online courses (Jan, 2015; Zimmerman and Kulikowich, 2016). Students with higher levels of online self-efficacy tend to spend more time using online learning technology and those students are more engaged in the learning process (Bates and Khasawneh, 2007; Chen, 2017), these effects being observed also during the COVID-19 pandemic. In uncertain, complex and ambiguous learning environments, strong self-efficacy beliefs contribute to more study engagement (Koob et al., 2021). In light of the above considerations, we expect that online self-efficacy has a positive relationship with learning engagement. Therefore, we predict that: *Online self-efficacy will positively predict learning engagement (H1)* (Figure 1).

### Learning Adaptability and Sources of Stress as Predictors of Engagement

Discussing predictive relations between relevant individual variables and learning engagement, for the specific purpose of our research, we selected the self-concept (in our study, expressed by online self-efficacy; self-regulation) and learning adaptability. Martin et al. (2013) found that adaptability, seen as cognitive, affective, and behavioral appropriate adjustments in uncertain, novel situations, significantly predicts academic (motivation, engagement, disengagement) and non-academic (self-esteem, life satisfaction, sense of meaning and purpose, emotional instability) outcomes. On the same note, Zhang et al. (2021) tested the relationship between adaptability and student engagement in the context of the COVID-19 pandemic, showing that adaptability directly increases student engagement. Regarding the relations between self-concept and learning engagement, Zhou et al. (2021) find that students’ self-concept clarity positively predicts learning engagement, through increased self-regulation.

Around the concept of individual adaptability, Ployhart and Bliese (2006) discuss the context-free nature of the construct, noting that individual adaptability is a reasonably stable, individual difference construct that influences how a person interprets and responds to different situations. In other words, individual adaptability allows a person to change her/his behavioral strategies in order to achieve different results despite the environmental features being the same. I-ADAPT theory refers to individual adaptability as having both direct and mediated influence on performance, the mediated effect occurring through the proximal mediating processes: situation perception and appraisal, strategy selection, self-regulation and coping, knowledge acquisition. Also, individual adaptability can take a proactive form, when a person anticipates changes in the environmental conditions and takes preventive measures to ensure accommodation, or a reactive form when the person responds to changes in the environment. The environment plays a moderating effect on the relationship between individual adaptability and performance (Ployhart and Bliese, 2006).

In our present study, the I-ADAPT theory was used to explain the prediction value of the individual adaptability on learning engagement and also support our hypothesis regarding the mediating action of self-regulation (as a proximal mediating process) on the effects of learning characteristics and sources of stress in relation to learning engagement.

Adaptability was investigated in many recent articles. Higher levels of academic adaptability are associated with lower levels of burnout and higher levels of learning engagement and academic performance (Xie et al., 2019). Studies conducted during the COVID-19 pandemic also showed that adaptability positively influence student engagement, supporting the behavioral function of adaptability as a propensity that helps individuals to adjust to the demands in their environment (Zhang et al., 2021). Given the fact that studies about the role of adaptability in students’ response to changes caused by COVID-19 are rare and starting from the evidence of previous studies we proposed the following hypothesis: *Learning adaptability will positively predict learning engagement (H2).*

The COVID-19 pandemic brought many challenges for students also on the academic dimension of their lives, university life has become more stressful than usual for many students (de la Fuente et al., 2021). Previous studies identified factors of academic stress pertaining to the teaching–learning process (such as maladjusted teaching methodology, poor classroom climate, and irrelevant content), factors related to the learning process including excessive learning activities, lack of control over one’s achievement (Cabanach et al., 2018; de la Fuente et al., 2021). These factors are even more prevalent in the context of the COVID-19 pandemic, recent research confirming that students experienced significantly higher levels of stress and isolation as well as negative mood and significantly lower levels of positive mood, relatedness, concentration and focus, motivation, and performance (Besser et al., 2020). The stress experienced by the student is an important predictor of their motivation and engagement (Salanova et al., 2009). Other theoretical models, such as the Demands-Resources-Theory (DR-T) (Bakker and de Vries, 2021) also explains that learning engagement is negatively affected by demands students face. During the COVID-19 pandemic, the academic demands were higher than ever and the effort and resources necessary to cope with them could lead to negative effects of these demands on study engagement, the results being confirmed by studies conducted on study engagement during the COVID-19 pandemic (Koob et al., 2021; Wester et al., 2021).

The transition from in-person to remote learning brought many challenges, such as the requirement to move off campus with little notice, inadequate learning spaces for learning while moving to their homes, technological difficulties, health- or economic-concerns, etc. (Wester et al., 2021). We expect to find a significant relation between stress and learning engagement, in accordance with (Schaufeli et al., 2002) research that negatively and significantly link engagement to burnout. Thus, we expect that stress emerging from different sources related to online learning experience during the COVID-19 pandemic to be a predictor, in a negative sense, for student engagement in learning. Therefore, we proposed the following hypothesis: *Sources of stress will negatively predict learning engagement (H3).*

### Self-Regulated Learning as Mediator

Self-regulated learning (SRL) can be seen as a form of intentional learning in which the learner has control over the process, steering, and directing cognitive and motivation processes to achieve the learning goal (Boekaerts and Cascallar, 2006). In this sense, self-regulated learning is a complex, strategic approach that involves multiple strategies used to set learning goals, deliberate, make decisions based on various factors. In this complex process, students who are able to better manage their emotions tend to be more efficient in self-regulating their learning and perform better at different learning tasks, whereas students who are inefficient at regulating their emotions are more inefficient in self-regulating their learning and therefore experience decreased learning achievement (Boekaerts and Cascallar, 2006). Self-regulated learning strategies refer to active approaches toward knowledge, engaging with the learning contents, using methods such as organizing and transforming information, self-consequating, seeking information, and rehearsing or using memory aids (Zimmerman, 1989).

The relation between academic achievement and self-regulated learning is also discussed by Barnard-Brak et al. (2010), who point out that there are different profiles of self-regulated learning behaviors, and those profiles can be associated with different learning outcomes. In terms of determinants (Zimmerman, 1989), discusses self-efficacy as one of the major factors that influence self-regulated learning, although the level of self-regulation varies depending on the specific context the learner finds herself in and is driven by one’s academic goals. Therefore, our including online self-efficacy as a determinant of self-regulated learning in online settings is sustained by previous research in the field. Self-regulated learning results from the interaction of personal, behavioral, and environmental, with self-regulated learning skills and strategies developing in an iterative manner, where students adjust their behaviors based on feedback and their evaluation regarding the advantages or disadvantages of the employed strategy (Barnard-Brak et al., 2010).

The SRL model that Barnard et al. (2009) use to determine student’s ability to self-regulate learning in learning environments that are fully or partially web-based focuses on specific behavioral approaches that students take toward learning, approaches that include: environment structuring, goal setting, time management, help seeking, task strategies, and self-evaluation. Students use these strategic processes in order to prepare for action (learning), control performance and reflect upon their learning experience and self-regulation efforts (Barnard-Brak et al., 2010).

Building upon the understanding that self-regulation in learning is associated with academic performance, it is noted that self-regulation is contextualized, meaning that the specific skills and strategies employed by the student are highly dependent on the learning context (Barnard-Brak et al., 2010). Therefore, it can be expected that self-regulation strategies used in a certain learning environment, such as face to face context, to be less effective in a different learning environment, such as the online context. While the positive association between self-efficacy and self-regulated learning is well established and much debated in the specialty literature, students with high self-efficacy being able to manage their time effectively, organize their work, minimize distractions, set goals for themselves, monitor their comprehension, ask for help when necessary, and maintain an effective work environment (Schunk et al., 2014), the associations between learning adaptability, academic stress and self-regulated learning are less tackled in current research. Previous studies showed that there is a positive association between self-regulation and study engagement (Wolters and Taylor, 2012), this relationship being observed also in online learning contexts (Xu and Qiu, 2021). Previous research has shown that self-regulation is an important predictor of engagement and also a significant outcome (Stefansson et al., 2018). While many studies investigated the effects of learning engagement on academic performance and dropout rates, self-regulation skills as antecedents or mediators of school engagement is not an extensively researched topic.

The shift to the remote emergency learning context generated many academic stressors that students had to manage. In order to maintain their involvement, they activated their self-regulated learning strategies which could act as mediators between the personal characteristics (such as online self-efficacy, learning adaptability, adaptability to uncertainty), academic stressors and learning engagement. More specifically, while online self-efficacy and adaptability will sustain the efficient use of self-regulated learning strategies which in turn, will lead to a higher learning engagement, the sources as stress will act as triggers for enabling SRL strategies as mechanism to cope with the stress in order to maintain higher levels of learning engagement. Therefore, we proposed the following hypotheses: *Self-regulated learning strategies will mediate the effects of learning characteristics and sources of stress on learning engagement (H4).*

## Materials and Methods

### Participants

The participants were 529 university students (M_age_ = 23.76), attending various study domains (social sciences, *N* = 66.5%, engineering, *N* = 22.5%, medicine, *N* = 7.8%, economics, *N* = 3.2%) from several universities in Romania, for different educational levels: first year students (*N* = 52.4%), second (*N* = 27%), third and fourth year of study (*N* = 20.6%).

### Instruments

The Online self-efficacy was measured through the Online self-efficacy scale (Gu et al., 2013). The 5 items had been adapted for the educational context and target self-evaluation as regards the capability in the use of technology and digital applications in the academic activity. The items have a high internal consistency, α = 0.93, had been measured on a 5-point Likert scale (1-to a very small extent, 5-to a very large extent).

Learning adaptability and Adaptability to uncertainty were measured through The Individual Learning Adaptability scale (Ployhart and Bliese, 2006). The items of the Learning Scale (7 items), and the Uncertainty Scale (9 items) had been adopted to reflect the individual adaptability to the academic context. A 5-point Likert scale (1− to a very small extent, 5− to a very large extent) was used. Cronbach’s Alpha for the Adaptability scale is 0.83 and for the Uncertainty scale is 0.82.

The sources of stress were measured through The Sources of Stress in Online Learning Scale (Authors, in press), the instrument which measures students’ perceived sources of stress associated with online learning. The 28 items are grouped into 6 dimensions: Inadequacy of teaching methods and teaching style—13 items (α = 0.92), Lack of social support—4 items (α = 0.81), Technical difficulties—3 items (α = 0.73), Role conflict—2 items (α = 0.60), Time constraints—2 items (α = 0.77), Diversity of techniques—2 items (α = 0.75), Inflexibility—2 items (α = 0.50). The measuring scale is a 5-point Likert scale (1− to a very small extent, 5− to a very large extent). Cronbach’s Alpha for the global SSOLS is 0.93. The Inflexibility scale was not included in the study given the low Cronbach’s Alpha value.

Self-regulated learning strategies were measured with the Online Self-Regulated Strategies Scale (OSLQ) (Barnard et al., 2009). According to the authors, the OSLQ is a 24-item scale with a 5-point Likert response format (values ranging from strongly agree to strongly disagree), higher scores being associated with better self-regulation in online learning. It consists of six subscales: environment structuring (α = 0.88), goal setting (α = 0.86), time management (α = 0.83), help seeking (α = 0.71); task strategies (α = 0.73), and self-evaluation (α = 0.80). The scores obtained from the measure demonstrated adequate internal consistency of scores with α = 0.91, indicating an adequate internal consistency (Barnard et al., 2009).

Learning engagement was measured with the UWES Learning Engagement Scale (Schaufeli et al., 2006). UWES assesses students’ academic engagement, and it is composed of 9 items that assess: vigor (3 items, α = 0.89), absorption (3 items, α = 0.74) and dedication (3 items, α = 0.80), each item is assessed using a Likert scale that ranges between 1—to a small extent and 5—to a very large extent. Cronbach’s Alpha for the total scale is 0.92.

### Procedure

A cross-sectional design was used. The previously described instruments were administered online during the second academic semester (May–June 2020), as self-report measures.

Our study followed the principles of the Declaration of Helsinki regarding ethical aspects on human subjects. We did not collect any data that could lead to the identification of the participants. The participants gave their informed consent, while the participation at the study was voluntary and no incentives were offered. The study was reviewed and approved by the Council of the Faculty of Psychology and Education Sciences, Transilvania University of Brasov, No 87/5.05.2020.

### Data Analysis

The normality assumption was met for all the numeric variables. The statistical design followed the cross-sectional research guidelines. Several significant associations between variables (Table 1) and the empirical support led us to a mediation model. The mediation hypothesis was tested using JASP 14.0. The statistical significance of meditation effects was assessed by interpreting the 95% bias-corrected confidence interval (5,000 samples). Given the fact that the variables are normally distributed, the estimation technique used was Maximum Likelihood (ML) A first order model was tested, to investigate the individual effects of each dimension on learning engagement. The individual factors (Online self-efficacy, Learning adaptability and Adaptability to uncertainty) and the six sources of stress (Inadequacy of teaching methods and teaching styles, Lack of social support, Technical difficulties, Role conflict, Time constraints and Diversity of techniques) were considered exogenous variables (predictors), while the online self-regulated learning strategies (Goal setting, Environment structuring, Task strategies, Time management, Help seeking, and Self-evaluation) were included as mediators and Learning engagement as criterion (predicted variable).

**TABLE 1:** Associations between the study variables.

Variables	1	2	3	4	5	6	7	8	9	10	11	12	13	14	15
1 Self-efficacy	—														
2 Engagement	0.289***	—													
3 Learning adaptability	0.326***	0.561***	—												
4 Adaptability to uncertainty	0.277***	0.328***	0.389***	—											
5 Goal setting	0.308***	0.599***	0.662***	0.315***	—										
6 Environment structuring	0.235***	0.423***	0.528***	0.158***	0.577***	—									
7 Task strategies	0.075	0.479***	0.527***	0.132***	0.543***	0.527***	—								
8 Time management	0.160***	0.473***	0.559***	0.219***	0.658***	0.545***	0.634***	—							
9 Help seeking	0.050	0.152***	0.166***	−0.033	0.235***	0.188***	0.294***	0.239***	—						
10 Self–evaluation	0.115**	0.418***	0.492***	0.193***	0.516***	0.418***	0.549***	0.507***	0.540***	—					
11 Inadequacy of teaching methods and teaching styles	−0.118**	−0.455***	−0.221***	−0.284***	−0.264***	−0.173***	−0.160***	−0.163***	0.055	−0.121**	—				
12 Lack of social support	−0.278***	−0.420***	−0.198***	−0.341***	−0.258***	−0.120**	−0.013	−0.131**	0.139**	−0.028	0.602***	—			
13 Technical difficulties	−0.437***	−0.160***	−0.088*	−0.177***	−0.136**	−0.105*	0.065	−0.045	0.010	0.011	0.294***	0.366***	—		
14 Role conflict	−0.149***	−0.206***	−0.053	0.010	−0.145***	−0.206***	−0.094*	−0.132**	0.001	−0.021	0.276***	0.233***	0.359***	—	
15 Time constraints	−0.084	−0.420***	−0.364***	−0.225***	−0.413***	−0.248***	−0.265***	−0.360***	0.046	−0.196***	0.408***	0.426***	0.202***	0.185***	—
16 Diversity of techniques	−0.153***	−0.094**	−0.022	−0.011	−0.100*	−0.054	0.055	0.022	0.032	0.098*	0.326***	0.209***	0.344***	0.357***	0.164***

N = 529, *p < 0.05, **p < 0.01, ***p < 0.001.

## Results

The Pearson coefficient correlations showed significant associations of the sources of stress in online learning with self-efficacy, leaning engagement, and self-regulated learning strategies (Table 1).

Including SRL strategies as mediators between the sources of stress and engagement, lead to a significant prediction. The model explained 54% of the total variance of learning engagement. The total variances explained for the self-regulated learning strategies were the following: Goal setting 49%, Environmental structuring 32%, Task strategies 34%, Time management strategies 35%, Help seeking 6%, and Self-evaluation 26%.

**FIGURE 2:**
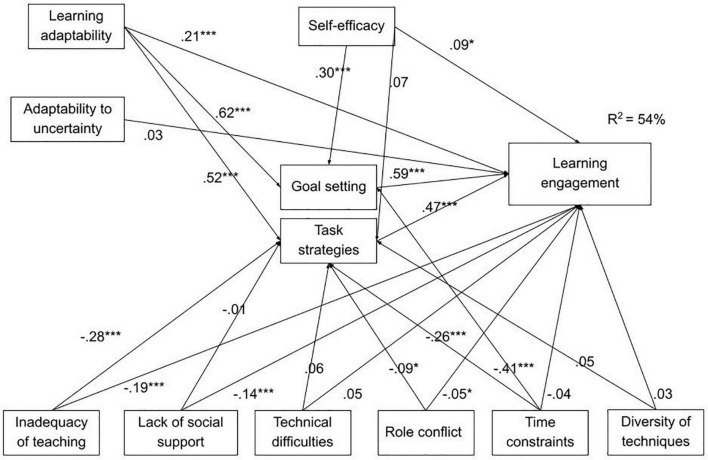
Path coefficients for the mediation model. *p < 0.05, ***p < 0.001.

The mediation model showed several significant direct and indirect effects of personal factors and sources of stress on learning engagement, confirming the hypothesis of a partial mediation (Table 2). The most significant direct effects were the following: self-efficacy and learning adaptability had positive direct effects on learning engagement (H1 and H2) and three sources of stress (Inadequacy of teaching methods and teaching styles, Lack of social support, and Role conflict) had negative effects (H3). The mediation hypotheses were sustained for several mediators: Goal setting strategies mediated both the association between online Self-efficacy and Learning engagement and between Learning adaptability and Engagement. Task strategies mediated the association between Learning adaptability and Engagement, all the others self-regulated learning strategies being not significant as mediators between individual factors and learning engagement. For the sources of stress, the results showed that Task strategies mediated the associations between all the six sources of stress and Learning engagement (H4) (Figure 2).

**TABLE 2:** Direct, indirect and total effects (significant) for the mediating effect of the self-regulated learning.

Effects on engagement	Predictor	Mediator	*B*	*Std. err*	β	*p*	95% CI	
							Lower	Upper
Direct	Self-efficacy		0.097	0.039	2.490	0.013	0.021	0.174
Indirect		Goal setting	0.024	0.010	2.381	0.017	0.004	0.044
Total			0.120	0.042	2.885	0.004	0.038	0.201
Direct	Learning adaptability		0.219	0.058	3.784	<0.001	0.106	0.332
Indirect		Goal setting	0.147	0.035	4.166	<0.001	0.078	0.216
Indirect		Task strategies	0.112	0.030	3.703	<0.001	0.053	0.171
Total			0.519	0.049	10.588	<0.001	0.423	0.615
Direct	Adaptability to uncertainty		0.035	0.051	0.684	0.494	−0.065	0.134
Total			−0.005	0.021	−0.234	0.815	−0.046	0.036
Direct	Inadequacy of teaching		−0.199	0.040	−5.002	<0.001	−0.277	−0.121
Indirect		Task strategies	−0.025	0.010	−2.473	0.013	−0.046	−0.005
Total			−0.239	0.042	−5.649	<0.001	−0.322	−0.156
Direct	Lack of social support		−0.144	0.035	−4.129	<0.001	−0.212	−0.076
Indirect		Task strategies	0.025	0.009	2.668	0.008	0.007	0.044
Total			−0.110	0.037	−3.011	0.003	−0.182	−0.039
Direct	Technical difficulties		0.056	0.037	1.536	0.124	−0.016	0.128
Indirect		Task strategies	0.018	0.009	2.055	0.040	0.001	0.035
Total			0.083	0.039	2.124	0.034	0.006	0.160
Direct	Role conflict		−0.054	0.027	−2.003	0.045	−0.107	−0.001
Indirect		Task strategies	−0.016	0.007	−2.380	0.017	−0.029	−0.003
Total			−0.082	0.028	−2.901	0.004	−0.138	−0.027
Direct	Time constraints		−0.047	0.030	−1.594	0.111	−0.105	0.011
Indirect		Goal setting	−0.030	0.009	−3.188	0.001	−0.048	−0.011
Indirect		Task strategies	−0.016	0.007	−2.280	0.023	−0.030	−0.002
Total			−0.093	0.031	−3.049	0.002	−0.154	−0.033
Direct	Diversity of techniques		0.038	0.029	1.272	0.203	−0.020	0.095
Indirect		Task strategies	0.015	0.007	2.131	0.033	0.001	0.029
Total			0.056	0.031	1.799	0.072	−0.005	0.117

*Standardized coefficients were computed. Only the significant effects were presented in this table. The values of all the paths are presented in Supplementary Material.*

## Discussion

The results of the study must be interpreted in the context in which at the beginning of the COVID-19 pandemic, universities from Romania decided unanimously to move didactic activities synchronously online and the use of ZOOM, Google Meet, Teams, and Skype communication platforms type, as well as the online educational platform Moodle type. The students participating in the study are enrolled in programs of study developed traditionally on-site, their experience being limited in asynchronous or hybrid online learning. In the context of COVID-19 pandemic, online learning engagement becomes an indicator of educational quality and a predictor of academic performance and of the dropout rate (Staikopoulos et al., 2015).

The study analyses the relation between individual characteristics (online self-efficacy, learning adaptability, and adaptability to uncertainty) and sources of stress and learning engagement in the online context through the forced transfer to online learning due to COVID-19 pandemic, as well as the role of online self-regulated learning strategies. According to Vayre and Vonthron (2017) learning engagement represents a psychological process having an important role in students’ course of study, it was an absolute necessity to identify the personal variables and the sources of stress which can determine it. The use of efficient self-regulated learning strategies, namely goal setting and task strategies could lead to a higher effect of self-efficacy and individual adaptability on engagement. In addition, the use of efficient self-regulated strategies could reduce the negative effects of sources of stress on learning engagement. The most important self-regulation strategies seemed to be goal setting and task strategies, which confirm the need for a clear structure of learning in the context of online activities. Through the proposed research model, we aim at offering a better understanding of personal mechanisms which influence online learning engagement in the pandemic context. Online self-efficacy, learning adaptability, and adaptability to uncertainty represent the individual characteristics which had been taken into consideration in the research model and had been related to learning engagement in the online learning context.

### Online Self-Efficacy and Learning Engagement

Online self-efficacy reflects students’ perception to use technical skills in order to achieve academic tasks as being a good predictor of learning engagement (Hu and Hui, 2012; Koob et al., 2021). The results of the study show that engagement in online learning rises if the value of online self-efficacy is also high. The instrument used by us in research underlines mainly self-efficacy related to the use of technology and web applications in the learning activity. Behavioral engagement in online learning activities is supported by personal convictions about having the abilities to use the digital tools specific to online learning. The relationship between online self-efficacy and learning engagement is positively low, according to the studies from the specialty literature (Lim, 2001; Hu and Hui, 2012; Vayre and Vonthron, 2017). Students with strong convictions about online self-efficacy have the tendency to make a greater effort and to persist in academic tasks (Schunk et al., 2014), manifesting adaptability, even in situations with a great level of uncertainty.

The positive association of online self- efficacy with learning engagement can be explained through the mechanism of the perceived behavioral control—a construct related to the individual’s perception of the availability of knowledge, resources, and opportunities required to perform the specific behavior (Venkatesh, 2000); the internal control over the personal knowledge and abilities necessary to succeed in performing tasks represent the behavioral anchor in using the necessary means to achieve specific academic tasks. The structure of internal control includes knowledge about technologies and applications (“I can get along well with technology and online applications”), and also self-efficacy in using them (“no matter how complex seems to be an application, I am sure I can understand it”), facilitating students’ engagement in the academic tasks specific to the online environment.

### Online Self-Efficacy and Self-Regulated Learning Strategies

The results of the study reveal positive associations between online self-efficacy and SRL strategies in the context of online learning, advocating the idea that self- efficacy promotes SRL behaviors (Pintrich, 1999), such as goal setting, environment structuring, time management, certifying previous studies (Joo et al., 2000; Artino and Jones, 2012). We observed that students who have a high value of online self-efficacy perceive themselves as being able to regulate efficiently the online learning processes. The results of the relations between variables do not bear the anticipated intensity but they are certainly suggestive. Learning strategies like task strategies—implies self-reflection strategies upon one’s own learning as well as the planning and implementation of correction strategies‘ and help seeking—pertaining to obtaining face to face clarifications from peers or teachers mediated by other means of communication, do not associate with online self-efficacy in the analyzed context. The effect of self-efficiency in online learning engagement is explained by the efficient use of self-regulated learning strategies. Among SRL strategies, the dimension goal setting explains the relation between online self -efficacy and online learning engagement. This might be due to the fact that, out of all the SRL strategies, goal setting is the first to be employed by the student (Zimmerman, 2000). Setting learning goals is rapidly triggered when faced with learning requirements and demands the less amount of time in comparison to other strategies such as time management or self-evaluation.

In our study, students filled out the research instruments at the beginning of their online learning experience caused by the pandemic. Given the novelty of the context (students did not experience online learning before) and based on that, from a SRL point of view, they were entering the first phase of the self-regulating process, as described by Zimmerman (2000): the forethought self-regulated learning stage that precede efforts to learn or perform (Zimmerman, 2000), in which they seek to identify the essential requirements of learning tasks, learning outcomes and set learning plans and objectives. During the forethought learning stage, students activate their beliefs upon self-efficacy, necessary to achieve learning tasks, with an impact over learning engagement (Cleary and Zimmerman, 2012). A high level of self-efficacy beliefs about using technical means necessary for online learning activity represents a motivator of online learning engagement and does not only have an adaptive role, through effort mobilization, persistence, commitment in the learning activity, but also it supports cognitive engagement in the efficient use of SRL strategies in learning. Thus, the relation between online self-efficacy and SRL strategies could be enhanced by experience in online learning activity according to Cleary and Zimmerman (2001) and Cleary and Zimmerman (2012), which opens new directions of research.

The speed and amplitude of the transition to online learning highlighted students’ adaptability to the new learning environment, this being an essential characteristic which allowed the use of new instruments and learning methods which therefore enabled online learning engagement. Online learning environment used by students during the COVID-19 pandemic can be described in terms of novelty, uncertainty and variability by also autonomy, which represented a real challenge for them (Martin et al., 2021). The online learning environment offers students the opportunity to control when, what, and how to study (Cunningham and Billingsley, 2003).

### Learning Adaptability and Learning Engagement

The results of our study highlights a direct positive relation between students’ capacity for adaptability and online learning engagement (Martin et al., 2013), and also an indirect positive relation, mediated by the efficient use of self-regulated learning strategies, namely goal setting, and task strategies. The students who demonstrated an adaptive capacity to comply with the uncertainty of the new learning environment manifested high levels of learning engagement, energy, and resistance during studies, even in difficult situations; they are strongly involved in their studies and experience a sense of significance, enthusiasm, inspiration, pride, and challenge, being fully concentrated and happily engrossed in what one is studying, where time passes quickly and one finds it difficult to detach him/herself from studying. The rapid and efficient adaption demonstrated students’ capacity to transfer what they learn in new learning situations but also the capacity to activate regulating strategies. This requires from students the self-evaluation of their own capacities and knowledge, selection of different strategies, necessary under the circumstances of online learning. Willingness to learn in a non-familiar environment, insecure and precarious represent a characteristic of adaptability in learning and implies the activation of those self-regulating strategies in all the stages of the learning process. Adaptability allowed students to engage in unfamiliar situations (online learning environment) efficiently and to activate self-regulated strategies, maintaining the control over uncertainty, diminishing as such the effect of stressors. Students who demonstrated high levels of adaptability assigned and followed their own learning objectives and also set task strategies, environment structuring, time management. Self-evaluations and help seeking proving learning engagement even in uncertainty situation. The study shows the fact that high levels of adaptability to uncertainty are reflected in positive reactions toward learning engagement, in the context of online learning.

The study demonstrates that adaptability represents a personal key resource (Ployhart and Bliese, 2006), a facilitator of engagement in different tasks, even in new situations implying uncertainty. Personal resources such as self-efficacy and adaptability, associated with efficient self-regulated strategies can lead to academic performances in online learning, a starting point which opens new directions of research.

The whole context determined by COVID-19 pandemic, characterized by insecurity and unpredictability represents a potential source of stress (Koolhaas et al., 2011). The sudden and enforced transition to online learning brought challenges students had to face as regards learning. Students’ learning engagement was influenced by isolation and social distance, by changes in the specific face-to-face learning routine, including environment conditions, by the use of technical means necessary for online learning which became a requisite. As we have demonstrated, certain personal characteristics (online self-efficacy, learning adaptability, and adaptability to uncertainty) facilitate learning engagement, being enhanced by the use of self-regulated efficient strategies in the context of online learning.

### Sources of Stress and Learning Engagement

Another objective of our study proposes to identify which sources of stress associated to online learning are predictors of online learning engagement and to which extent self-regulated learning strategies mediate the relation. Between the measured sources of stress and online learning engagement we identified significant negative relations with different levels of intensity. Between sources of stress associated to the use of technical tools and online learning engagement significant negative relations have been measured, but are of low intensity. The category of technological difficulties refers to the constraints caused by the infrastructure offered by universities and its functioning, the devices used by students, the limited/difficult access to internet sources and the category of diversity of techniques refers to simultaneous use of different tools specific to digital learning (microphone, video, chat, sharing materials etc.). Technical difficulties, respectively, diversity of techniques represent sources of stress which can interfere with online learning activity, through a rise in the number of disruptions, which may generate a rupture in the cognitive focus, cognitive interferences, setbacks in processing information and in increasing effort, preventing thereby the progress in learning tasks (Jett and George, 2003). Thus, the cognitive task of learning is perceived as having a high level of difficulty, which can lead to non-engagement in learning. Low levels of technical difficulties perception, respectively, of diversity of techniques are associated with high levels of online learning engagement, while high levels of online self-efficacy are associated negatively with technical difficulties perception, respectively, with diversity of techniques, which confirms the fact that there is variability in the modality of students’ answers. The more recent literature on this topic (Kostaki and Karayianni, 2021) points out that self- efficacy moderates the relation between technical difficulties and learning engagement, therefore students who were not confident in their computer skills and encountered technical difficulties had lower engagement scores than those with higher online self-efficacy facing a similar situation, which opens new directions in research.

Instruction environments, respectively, online learning environments are considered instruments, which, designed efficiently, offer numerous gains both in teaching and in learning. Students have the possibility to engage actively in learning, reflecting critically upon the information, which facilitate the acquisition of knowledge and metacognitive abilities, while teachers have the possibility to address to a large community of students and to transform teacher-centered passive instruction, to active instruction, student-oriented, through reconsidering the instructional design and the teaching style. The sudden transition to online instructional environment obliged teachers to adapt their design and teaching style rapidly to the virtual environment instructional activity.

Significant relative relations have been found between time constraints and online learning engagement. Research on time constraints in online learning are parochial. The perception of time constraints represents a source of stress, which affects learning activity, task engagement, and task performance. Students who perceive a lower pressure of time have are lucky enough to be self-determined in order to engage in learning tasks. The results of our study point out a significant negative relation between inadequacy of teaching methods and teaching styles and online learning engagement, relation supported by current studies developed in similar contexts (Yao et al., 2020; Li et al., 2021). Among the causes identified are teachers’ unfamiliarity and lack of experience in approaching online learning platforms, teachers’ lack of experience in developing/transforming the educational content for the online activity. In order to facilitate students’ engagement in the learning activity, the teacher must use methods based on research and exploration, on students’ interaction, which support reasoning, critical thinking and high rank cognitive activities (Hannel and Hannel, 1998). Also, the teaching style adapted to virtual environment is based on the use of several channels of transmitting information, on the activation of the group of students, and on a careful and steady monitoring of the learning activities, materialized through the offer of a prompt, adequate, and rapid feedback which would help students maintain interest and sustain their effort in the learning tasks. Through an adequate use of instruction methods and of a learning style adapted to the virtual environment, self-regulated learning, critical thinking and sustainable engagement to online learning can be improved (Li et al., 2021). In order to sustain the quality of online learning engagement it would be useful to identify the characteristics of the instructional design and the teaching style specific to the synchronous online instructional environment in subsequent studies.

The significantly negative relation, proved by the study between the lack of social support and online learning engagement is also supported by other studies carried out in the online context (Zhang et al., 2020; Oliveras-Ortiz et al., 2021). Social relation established between teachers and students, students and students, even mediated by communication means are perceived to contribute to the social support necessary to engagement in learning tasks. The main source of social support which contributes to online learning engagement is the teacher (Czerkawski and Lyman, 2016). The teacher encourages, offers adequate, real-time feedback, perceived as a motivating factor of learning engagement.

Role conflict appears as an effect of the tensions created between personal life and professional life. Role conflict occurs when an individual perceives incompatible time, strain, or behavior-based demands between work and non-work roles. Role conflict appears as a source of stress associated to learning engagement. In the specialty literature the role conflict associated to online learning context is insufficiently studied as compared to working context. The studies showed a significant positive association between distance working done at home and work-to-family conflict. Difficulty separating work and family spheres and fully unplugging from work can increase role conflict for teleworkers (Carnevale and Hatak, 2020). Online learning which was usually done from home, interfered with family responsibilities and other roles, being a potential generator source of conflict.

### Self-Regulated Learning Strategies, Learning Characteristics, Sources of Stress, and Learning Engagement

Self-regulated strategies—task strategies and goal setting represent mediators of stressors perceived by the students under the conditions of the sudden transition to online activity and online learning engagement. Task strategies represents the regulating mechanisms which diminish the negative effect of the analyzed sources of stress—inadequacy of teaching methods and teaching styles, lack of social support, technical difficulties, role conflict, time constraint, and diversity of techniques), and goal setting intervenes additionally diminishing the effect time constraint over online learning engagement. The regulation of the learning environment plays an important role in students’ learning (Pintrich, 2004), thus in online environment students are determined to use self-regulated strategies which need the planning and regulation of time and environment and more effort in seeking help from peers and teachers. Students demonstrated self-control behaviors which imply goal setting and task strategies, specific structuring mechanisms of the new learning environment.

### Limitations and Implications

The findings of this study should be seen in light of some limitations. A first limitation refers to the low Cronbach’s Alpha values for some of the Sources of Stress in Online Learning Scale, specifically for the Role conflict scale, a possible explanation being the small number of items included in this scale. We also dropped the Inflexibility scale which had a low reliability. Further studies should investigate alternative factor structures of the instrument and focus also on other psychometric properties such as predictive validity. A second limitation concerns the use of self-report measures which imply the possibility of biased answers because of the participants’ report on their own experiences and the cross-sectional design (the instruments were administered all at once) which do not allow to draw causal conclusions. A follow-up study as a future research direction could offer a dynamic view on the evolution of students; perceptions, use of self-regulation strategies and management of the sources of stress and their impact on learning engagement.

A third limit refers to the convenience sampling that was used in this study. Participants were not randomly selected to take part in the study, thus the sample may lack proper representation. Given the nature of the psychosocial context at the time of the study (social anxiety and the subsequent restrictions regarding direct, face to face interactions; presumably low psychological availability to engage in actions such as responding to lengthy questionnaires in various research studies), getting students to thoroughly participate in our research was not as easy as to make us comfortable in a position of probabilistically selection. However, we do acknowledge the possible biased results stemming from not reaching participants based on balanced representation but based on voluntary choice. We are also aware of the limitation of not being able to generalize our present results beyond the studied sample.

The implications of this study reside in the increased awareness regarding how learning engagement in online learning can be predicted by individual characteristics such as online self-efficacy and learning adaptability, with statistically discernible effects of several sources of stress such as inadequacy of teaching methods and styles, lack of social support, and role conflict, and the mediating influence of self-regulated strategies such as Task strategies, Goal setting on the relationship between individual characteristics and learning engagement. Therefore, from a pedagogical standpoint, it can prove useful for an educator to consider using teaching strategies and designing learning opportunities that foster specific learning practices in students enrolled in university programs, learning practices that relate to setting feasible yet challenging learning goals and striving toward them, and systematically self-assigning and performing learning tasks pertaining to their learning goals. From a psychological point of view, studying the relationship between various perceived sources of stress related to the academic experience, such as lack of social support or role conflict, and learning related behaviors (ex. Task strategies) can be a step forward in better understanding the coping mechanisms that young adults—with different levels of self-efficacy and learning adaptability- employ in dealing with the specific challenges they face.

## Conclusion

The main aim of the present research is to analyze the predictive value of individual characteristics such as online self-efficacy, adaptability to uncertainty and sources of stress during online learning on learning engagement. We also aimed to highlight if these relationships could be mediated by the online self-regulated learning strategies, during the COVID-19 pandemic.

The use of efficient self-regulated learning strategies, namely goal setting, and task strategies could lead to a higher effect of self-efficacy and individual adaptability on engagement. Furthermore, the use of efficient self-regulated strategies could reduce the negative effects of sources of stress on learning engagement. The most important self-regulation strategies seemed to be goal setting and task strategies, which confirm the need for a clear structure of learning in the context of online activities. Our study explores possible antecedents of online learning engagement, stress factors and self-regulated mechanisms in the context of the sudden change of the learning environment during COVID-19 pandemic.

## Data Availability Statement

The raw data supporting the conclusions of this article will be made available by the authors, without undue reservation.

## Ethics Statement

The studies involving human participants were reviewed and approved by the Council of the Faculty of Psychology and Education Sciences, Transilvania University of Brasov, No 87/5.05.2020. The patients/participants provided their written informed consent to participate in this study.

## Author Contributions

IRT and DVN developed the study concept. MMS conducted the data collection and drafted the manuscript. A-MC performed the data analysis. All authors took part in result interpretation, reviewed, edited several versions of the manuscript, provided critical revisions, and approved the final version of the manuscript for submission.

## Conflict of Interest

The authors declare that the research was conducted in the absence of any commercial or financial relationships that could be construed as a potential conflict of interest.

## Publisher’s Note

All claims expressed in this article are solely those of the authors and do not necessarily represent those of their affiliated organizations, or those of the publisher, the editors and the reviewers. Any product that may be evaluated in this article, or claim that may be made by its manufacturer, is not guaranteed or endorsed by the publisher.
