# Supplementary material for: Predictors of Learning Engagement in the Context of Online Learning During the COVID-19 Pandemic
Source: Front Psychol. 2022 Apr 29;13:867122. doi: 10.3389/fpsyg.2022.867122 (PMC9100394; doi:10.3389/fpsyg.2022.867122)
Supplement: Supplementary file 1 [file Table_1.DOCX]

Supplementary Material

# Direct, indirect and total effects for the mediating effect of the self-regulated learning (Parameter estimates, standardized coefficients)

|  | | | | | | | | | | | | | | | | | | | | |
| --- | --- | --- | --- | --- | --- | --- | --- | --- | --- | --- | --- | --- | --- | --- | --- | --- | --- | --- | --- | --- |
|  | | | | | | | | | | | | | | | | 95% Confidence Interval | | | | |
| **Direct effects** |  | |  | | *B* | | | *St. err* | | | *β* | | *p* | | Lower | | | Upper | |  |
| Self-efficacy |  | → |  | Learning engagement |  | 0.097 |  | | 0.039 |  | | 2.490 |  | 0.013 |  | 0.021 |  | | 0.174 |  |
| Learning adaptability |  | → |  | Learning engagement |  | 0.219 |  | | 0.058 |  | | 3.784 |  | < .001 |  | 0.106 |  | | 0.332 |  |
| Adaptability to uncertainty |  | → |  | Learning engagement |  | 0.035 |  | | 0.051 |  | | 0.684 |  | 0.494 |  | -0.065 |  | | 0.134 |  |
| Inadequacy of teaching |  | → |  | Learning engagement |  | -0.199 |  | | 0.040 |  | | -5.002 |  | < .001 |  | -0.277 |  | | -0.121 |  |
| Lack of social support |  | → |  | Learning engagement |  | -0.144 |  | | 0.035 |  | | -4.129 |  | < .001 |  | -0.212 |  | | -0.076 |  |
| Technical difficulties |  | → |  | Learning engagement |  | 0.056 |  | | 0.037 |  | | 1.536 |  | 0.124 |  | -0.016 |  | | 0.128 |  |
| Role conflict |  | → |  | Learning engagement |  | -0.054 |  | | 0.027 |  | | -2.003 |  | 0.045 |  | -0.107 |  | | -0.001 |  |
| Time constraints |  | → |  | Learning engagement |  | -0.047 |  | | 0.030 |  | | -1.594 |  | 0.111 |  | -0.105 |  | | 0.011 |  |
| Diversity of techniques |  | → |  | Learning engagement |  | 0.038 |  | | 0.029 |  | | 1.272 |  | 0.203 |  | -0.020 |  | | 0.095 |  |
|  | | | | | | | | | | | | | | | | | | | | |
|  | | | | | | | | | | | | | | | | | | | | |

| **Indirect effects on** | | | | | | | | | | | | | | | | | | | | | | | | | 95% Confidence  Interval | | | | |  |
| --- | --- | --- | --- | --- | --- | --- | --- | --- | --- | --- | --- | --- | --- | --- | --- | --- | --- | --- | --- | --- | --- | --- | --- | --- | --- | --- | --- | --- | --- | --- |
| → **Learning engagement** |  | | | **Mediator** | | |  | | |  | | | *B* | | | *St. err* | | | *β* | | | *p* | | | Lower | | | Upper | |  |
| Self-efficacy |  | → |  | | Goal setting |  | |  |  | |  |  | | 0.024 |  | | 0.010 |  | | 2.381 |  | | 0.017 |  | | 0.004 |  | | 0.044 |  |
|  |  | → |  | | Environ. structuring |  | |  |  | |  |  | | -3.322e -4 |  | | 0.004 |  | | -0.093 |  | | 0.926 |  | | -0.007 |  | | 0.007 |  |
|  |  | → |  | | Task strategies |  | |  |  | |  |  | | -0.002 |  | | 0.008 |  | | -0.277 |  | | 0.782 |  | | -0.018 |  | | 0.013 |  |
|  |  | → |  | | Time management |  | |  |  | |  |  | | -4.485e -5 |  | | 5.215e -4 |  | | -0.086 |  | | 0.931 |  | | -0.001 |  | | 9.772e -4 |  |
|  |  | → |  | | Help seeking |  | |  |  | |  |  | | 7.804e -4 |  | | 0.002 |  | | 0.437 |  | | 0.662 |  | | -0.003 |  | | 0.004 |  |
|  |  | → |  | | Self-evaluation |  | |  |  | |  |  | | -1.237e -4 |  | | 0.003 |  | | -0.039 |  | | 0.969 |  | | -0.006 |  | | 0.006 |  |
| Learning adaptability |  | → |  | | Goal setting |  | |  |  | |  |  | | 0.147 |  | | 0.035 |  | | 4.166 |  | | < .001 |  | | 0.078 |  | | 0.216 |  |
|  |  | → |  | | Environ. structuring |  | |  |  | |  |  | | -0.002 |  | | 0.025 |  | | -0.093 |  | | 0.926 |  | | -0.052 |  | | 0.048 |  |
|  |  | → |  | | Task strategies |  | |  |  | |  |  | | 0.112 |  | | 0.030 |  | | 3.703 |  | | < .001 |  | | 0.053 |  | | 0.171 |  |
|  |  | → |  | | Time management |  | |  |  | |  |  | | -0.003 |  | | 0.028 |  | | -0.092 |  | | 0.926 |  | | -0.058 |  | | 0.053 |  |
|  |  | → |  | | Help seeking |  | |  |  | |  |  | | 0.007 |  | | 0.011 |  | | 0.681 |  | | 0.496 |  | | -0.014 |  | | 0.029 |  |
|  |  | → |  | | Self-evaluation |  | |  |  | |  |  | | 0.039 |  | | 0.027 |  | | 1.464 |  | | 0.143 |  | | -0.013 |  | | 0.092 |  |
| Adaptability to uncertainty |  | → Goal setting | | | | | | | | | |  | | 0.009 |  | | 0.011 |  | | 0.797 |  | | 0.425 |  | | -0.013 |  | | 0.031 |  |
|  |  | → Environ.structuring | | | | | | | | | |  | | 3.213e -4 |  | | 0.003 |  | | 0.093 |  | | 0.926 |  | | -0.006 |  | | 0.007 |  |
|  |  | → Task strategies | | | | | | | | | | | | -0.013 |  | | 0.011 |  | | -1.237 |  | | 0.216 |  | | -0.034 |  | | 0.008 |  |
|  |  | → |  | | Time management | | | | | | | | | -3.962e -5 |  | | 4.948e -4 |  | | -0.080 |  | | 0.936 |  | | -0.001 |  | | 9.301e -4 |  |
|  |  | → |  | | Help seeking | | | | | | |  | | -0.003 |  | | 0.004 |  | | -0.626 |  | | 0.531 |  | | -0.011 |  | | 0.006 |  |
|  |  | → |  | | Self-evaluation | | | | | | | | | 0.002 |  | | 0.004 |  | | 0.399 |  | | 0.690 |  | | -0.007 |  | | 0.010 |  |
| Inadequacy of teaching |  | → |  | | Goal setting | | | | | | |  | | -0.007 |  | | 0.009 |  | | -0.742 |  | | 0.458 |  | | -0.024 |  | | 0.011 |  |
|  |  | → |  | | Environ. t structuring | | | | | | |  | | 2.089e -4 |  | | 0.002 |  | | 0.093 |  | | 0.926 |  | | -0.004 |  | | 0.005 |  |
|  |  | → Task strategies | | | | | | | | | | | | -0.025 |  | | 0.010 |  | | -2.473 |  | | 0.013 |  | | -0.046 |  | | -0.005 |  |
|  |  | → |  | | Time management | | | | | |  |  | | 5.360e -5 |  | | 6.117e -4 |  | | 0.088 |  | | 0.930 |  | | -0.001 |  | | 0.001 |  |
|  |  | → | Help seeking | | | | | | | | | | | -8.316e -4 |  | | 0.002 |  | | -0.449 |  | | 0.654 |  | | -0.004 |  | | 0.003 |  |
|  |  | → |  | | Self-evaluation | | | | | | |  | | -0.007 |  | | 0.006 |  | | -1.221 |  | | 0.222 |  | | -0.018 |  | | 0.004 |  |
| Lack of social support |  | → |  | | Goal setting | | | | | | | | | -0.002 |  | | 0.008 |  | | -0.324 |  | | 0.746 |  | | -0.017 |  | | 0.012 |  |
|  |  | → |  | | Environ. structuring | | | | | | |  | | -2.038e -4 |  | | 0.002 |  | | -0.093 |  | | 0.926 |  | | -0.005 |  | | 0.004 |  |
|  |  | → |  | | Task strategies | | | | | | |  | | 0.025 |  | | 0.009 |  | | 2.668 |  | | 0.008 |  | | 0.007 |  | | 0.044 |  |
|  |  | → Time management | | | | | | | | | |  | | -2.050e -4 |  | | 0.002 |  | | -0.092 |  | | 0.927 |  | | -0.005 |  | | 0.004 |  |
|  |  | → Help seeking | | | | | | | | | |  | | 0.004 |  | | 0.006 |  | | 0.672 |  | | 0.502 |  | | -0.007 |  | | 0.014 |  |
|  |  | → Self-evaluation | | | | | | | | | |  | | 0.007 |  | | 0.006 |  | | 1.285 |  | | 0.199 |  | | -0.004 |  | | 0.018 |  |
| Technical difficulties |  | → Goal setting | | | | | | | | | |  | | 0.009 |  | | 0.008 |  | | 1.089 |  | | 0.276 |  | | -0.007 |  | | 0.025 |  |
|  |  | → Environ. structuring | | | | | | | | | | | | -6.520e 5 |  | | 7.210e -4 |  | | -0.090 |  | | 0.928 |  | | -0.001 |  | | 0.001 |  |
|  |  | → Task strategies | | | | | | | | | |  | | 0.018 |  | | 0.009 |  | | 2.055 |  | | 0.040 |  | | 8.227e -4 |  | | 0.035 |  |
|  |  | → Time management | | | | | | | | | |  | | -1.644e -4 |  | | 0.002 |  | | -0.092 |  | | 0.927 |  | | -0.004 |  | | 0.003 |  |
|  |  | → Help seeking | | | | | | | | | | | | -9.613e -4 |  | | 0.002 |  | | -0.504 |  | | 0.614 |  | | -0.005 |  | | 0.003 |  |
|  |  | → Self-evaluation | | | | | | | | | | | | 0.001 |  | | 0.003 |  | | 0.359 |  | | 0.719 |  | | -0.005 |  | | 0.007 |  |
| Role conflict |  | → Goal setting | | | | | | | | | | | | -0.011 |  | | 0.006 |  | | -1.679 |  | | 0.093 |  | | -0.023 |  | | 0.002 |  |
|  |  | → Environ.structuring | | | | | | | | | | | | 5.176e -4 |  | | 0.006 |  | | 0.093 |  | | 0.926 |  | | -0.010 |  | | 0.011 |  |
|  |  | → Task strategies | | | | | | | | | |  | | -0.016 |  | | 0.007 |  | | -2.380 |  | | 0.017 |  | | -0.029 |  | | -0.003 |  |
|  |  | → Time management | | | | | | | | | | | | 4.111e -4 |  | | 0.004 |  | | 0.092 |  | | 0.926 |  | | -0.008 |  | | 0.009 |  |
|  |  | → Help seeking | | | | | | | | | |  | | -4.115e -4 |  | | 0.001 |  | | -0.368 |  | | 0.713 |  | | -0.003 |  | | 0.002 |  |
|  |  | → Self-evaluation | | | | | | | | | | | | -0.002 |  | | 0.003 |  | | -0.816 |  | | 0.414 |  | | -0.007 |  | | 0.003 |  |
| Time constraints |  | → Goal setting | | | | | | | | | |  | | -0.030 |  | | 0.009 |  | | -3.188 |  | | 0.001 |  | | -0.048 |  | | -0.011 |  |
|  |  | → Environ. structuring | | | | | | | | | | | | 1.618e -4 |  | | 0.002 |  | | 0.093 |  | | 0.926 |  | | -0.003 |  | | 0.004 |  |
|  |  | → Task strategies | | | | | | | | | |  | | -0.016 |  | | 0.007 |  | | -2.280 |  | | 0.023 |  | | -0.030 |  | | -0.002 |  |
|  |  | → Time management | | | | | | | | | | | | 6.666e -4 |  | | 0.007 |  | | 0.092 |  | | 0.926 |  | | -0.013 |  | | 0.015 |  |
|  |  | → Help seeking | | | | | | | | | |  | | 0.001 |  | | 0.002 |  | | 0.602 |  | | 0.547 |  | | -0.003 |  | | 0.005 |  |
|  |  | → Self-evaluation | | | | | | | | | |  | | -0.003 |  | | 0.003 |  | | -0.900 |  | | 0.368 |  | | -0.008 |  | | 0.003 |  |
| Diversity of techniques |  | → Goal setting | | | | | | | | | |  | | -0.004 |  | | 0.006 |  | | -0.613 |  | | 0.540 |  | | -0.017 |  | | 0.009 |  |
|  |  | → Environ. structuring | | | | | | | | | |  | | -1.288e -4 |  | | 0.001 |  | | -0.092 |  | | 0.926 |  | | -0.003 |  | | 0.003 |  |
|  |  | → Task strategies | | | | | | | | | |  | | 0.015 |  | | 0.007 |  | | 2.131 |  | | 0.033 |  | | 0.001 |  | | 0.029 |  |
|  |  | → Time management | | | | | | | | | | | | -3.229e -4 |  | | 0.003 |  | | -0.092 |  | | 0.926 |  | | -0.007 |  | | 0.007 |  |
|  |  | → Help seeking | | | | | | | | | |  | | 5.651e -4 |  | | 0.001 |  | | 0.428 |  | | 0.669 |  | | -0.002 |  | | 0.003 |  |
|  |  | → Self-evaluation | | | | | | | | | |  | | 0.007 |  | | 0.006 |  | | 1.337 |  | | 0.181 |  | | -0.003 |  | | 0.018 |  |
|  | | | | | | | | | | | | | | | | | | | | | | | | | | | | | |  |

|  | | | | | | | | | | | | | | | | | | | |
| --- | --- | --- | --- | --- | --- | --- | --- | --- | --- | --- | --- | --- | --- | --- | --- | --- | --- | --- | --- |
|  | | | | | | | | | | | | | | | 95% Confidence  Interval | | | | |
| **Total effects** |  | |  | | *B* | | *Std. err* | | | *β* | | *p* | | Lower | | | Upper | |  |
| Self-efficacy |  | → |  | Learning engagement |  | 0.120 |  | 0.042 |  | | 2.885 |  | 0.004 |  | 0.038 |  | | 0.201 |  |
| Learning adaptability |  | → |  | Learning engagement |  | 0.519 |  | 0.049 |  | | 10.588 |  | < .001 |  | 0.423 |  | | 0.615 |  |
| Adaptability to uncertainty |  | → |  | Learning engagement |  | 0.030 |  | 0.054 |  | | 0.551 |  | 0.582 |  | -0.076 |  | | 0.135 |  |
| Inadequacy of teaching |  | → |  | Learning engagement |  | -0.239 |  | 0.042 |  | | -5.649 |  | < .001 |  | -0.322 |  | | -0.156 |  |
| Lack of social support |  | → |  | Learning engagement |  | -0.110 |  | 0.037 |  | | -3.011 |  | 0.003 |  | -0.182 |  | | -0.039 |  |
| Technical difficulties |  | → |  | Learning engagement |  | 0.083 |  | 0.039 |  | | 2.124 |  | 0.034 |  | 0.006 |  | | 0.160 |  |
| Role conflict |  | → |  | Learning engagement |  | -0.082 |  | 0.028 |  | | -2.901 |  | 0.004 |  | -0.138 |  | | -0.027 |  |
| Time constraints |  | → |  | Learning engagement |  | -0.093 |  | 0.031 |  | | -3.049 |  | 0.002 |  | -0.154 |  | | -0.033 |  |
| Diversity of techniques |  | → |  | Learning engagement |  | 0.056 |  | 0.031 |  | | 1.799 |  | 0.072 |  | -0.005 |  | | 0.117 |  |
|  | | | | | | | | | | | | | | | | | | | |
| Note.  Delta method standard errors, normal theory confidence intervals, ML estimator. | | | | | | | | | | | | | | | | | | | |
